# Global Dynamic Path Planning of AGV Based on Fusion of Improved A* Algorithm and Dynamic Window Method

**DOI:** 10.3390/s24062011

**Published:** 2024-03-21

**Authors:** Te Wang, Aijuan Li, Dongjin Guo, Guangkai Du, Weikai He

**Affiliations:** 1 School of Automotive Engineering, Shandong Jiaotong University, Jinan 250357, China; 17861403495@163.com; 2 Research and Development Department, Shandong Huali Electromechanical Co., Ltd., Jining 250101, China; hl7060777@126.com; 3 School of Aeronautics, Shandong Jiaotong University, Jinan 250357, China; jiandanlcc@163.com

**Keywords:** AGV, improved A* algorithm, dynamic window method, dynamic obstacle avoidance

## Abstract

Designed to meet the demands of AGV global optimal path planning and dynamic obstacle avoidance, this paper proposes a combination of an improved A* algorithm and dynamic window method fusion algorithm. Firstly, the heuristic function is dynamically weighted to reduce the search scope and improve the planning efficiency; secondly, a path-optimization method is introduced to eliminate redundant nodes and redundant turning points in the path; thirdly, combined with the improved A* algorithm and dynamic window method, the local dynamic obstacle avoidance in the global optimal path is realized. Finally, the effectiveness of the proposed method is verified by simulation experiments. According to the results of simulation analysis, the path-planning time of the improved A* algorithm is 26.3% shorter than the traditional A* algorithm, the search scope is 57.9% less, the path length is 7.2% shorter, the number of path nodes is 85.7% less, and the number of turning points is 71.4% less. The fusion algorithm can evade moving obstacles and unknown static obstacles in different map environments in real time along the global optimal path.

## 1. Introduction

With the wide use of automatic guided vehicles (AGVs) in logistics, warehousing and industrial production, its path-planning technology has been widely studied by scholars. The path planning of AGV refers to the generation of a safe, collision-free path based on the shortest time or the shortest path in a map environment. The effect of path planning will determine the efficiency, safety and stability of AGV operation. From the degree of mastery of the operating environment, the path planning of AGVs includes global path planning and local path planning [1]. The global path planning of AGV is to generate the global optimal path by using the search algorithm to avoid static obstacles in the known work scene. The commonly used global path-planning algorithms include graph-based search algorithms (such as Dijkstra algorithm [2], A* algorithm [3,4,5,6,7,8]), sampling-based algorithms (such as rapidly exploring random tree algorithm [9]) and intelligent algorithms (such as genetic algorithm [10], ant colony algorithm [11,12]). Local path planning means that AGVs use sensors to collect local environment information for real-time dynamic obstacle avoidance during operation. The dynamic window method [13] and artificial potential field method [14] are often used as local path-planning algorithms.

The A* algorithm, since it has the advantages of a simple structure and a small amount of calculation, is widely applied to grid map global path planning. However, there are many drawbacks in the traditional A* algorithm, such as low search efficiency in a complex environment, a lot of redundant nodes and turning points in the path and an unsmooth path. Wang et al. [3] reduced the number of turns by introducing the length of the planned completed path and the time cost required for the planned completed path into the cost function of the A* algorithm, and then removed the nodes passing through the vertices of the obstacle and adopted the arc turning strategy to generate a safe and smooth AGV path. Lai et al. [4] reduced the expansion nodes and shorten the search time by improving the neighborhood search strategy and heuristic function, deleted redundant nodes by introducing a method of preserving key nodes, and combined with a piecewise second-order Bezier curve to generate the smooth path for mobile robot driving. Zhang et al. [5] introduced a deviation factor in the heuristic function to reduce the total number of expansion nodes, which is the vertical distance between the current node and the connection of starting and target point, used the bi-directional A* search strategy to speed up path search, and combined with B-spline curve. The path meeting the needs of surface unmanned vehicle is then generated. Zheng et al. [6] added the hop search strategy on the traditional A* algorithm to speed up the search speed of the path node of the A* algorithm, and used the angle evaluation to reduce the quantity of turning points so as to enhance the running efficiency of AGVs. Fang et al. [7] introduced a logarithmic attenuation factor into heuristic function, proposed the strategy of solving key nodes, reduced the search nodes and path turning points, and integrated the A* algorithm with dynamic window method which solved the problems that the path of the A* algorithm cannot meet the driving demand of AGVs and the dynamic window method cannot reach the target point. Although the above literature has made different improvements to the A* algorithm, it still does not solve the problem of dynamic obstacle avoidance. Kim et al. [13] proposed an algorithm that combines the dynamic window method and deep reinforcement learning to achieve local dynamic obstacle avoidance, but this algorithm is not suitable for global optimal path planning. Yang et al. [15] proposed a global dynamic path-planning method which combines an ant colony algorithm and the dynamic window method, but the efficiency of this algorithm needs to be improved.

Although the A* algorithm has strong global path-planning ability, its planned path is not smooth and cannot achieve dynamic obstacle avoidance. The dynamic window method has a good ability for local path planning, which can achieve dynamic obstacle avoidance, but it is not suitable for global path planning. In this paper, a path-planning fusion algorithm is proposed to achieve the global optimal path planning of AGVs, improve the efficiency of path planning, and achieve real-time dynamic obstacle avoidance. Firstly, by improving the cost function of the traditional A* algorithm, the path search is more directional, the expansion of nodes is lower and the search time is shorter. Secondly, by using path optimization strategy to eliminate redundant nodes and redundant turning points in the global path, the path length is shortened and the path smoothness is improved. Finally, the fusion of the global planning capability of the A* algorithm and the local planning capability of the dynamic window method enables the AGV to realize dynamic obstacle avoidance in global path planning, effectively avoiding dynamic obstacles and unknown static obstacles that may appear in the driving path.

## 2. Global Path Planning Based on Improved A* Algorithm

### 2.1. Traditional A* Algorithm

The traditional A* algorithm is a heuristic search algorithm developed on the basis of the Dijkstra algorithm. Compared with the blind search of the Dijkstra algorithm, traditional A* algorithms incorporate heuristic functions in the neighborhood search process to provide search directions for path planning [8]. After the heuristic function is included, the cost function is used to evaluate the neighborhood nodes around the starting point, select the least costly node as the next node in the surrounding neighborhood, then evaluate around the neighborhood nodes of the current node, and so on until the target point is searched. The traditional A* algorithm calculates the cost value as follows:

(1) f(n)=g(n)+h(n)

In the formula, f(n) is the estimated value from the starting point to the target point, g(n) is the actual value from the starting point to the node n, h(n) is the estimated value from node *n* to the target point.

In the paper, we use the Euclidean distance for the actual cost value g(n) and the Chebyshev distance for the estimated cost value h(n). The calculation formulas are as follows:

(2) $$g(n)=\sqrt{(x_{n}-x_{s}{)}^{2}+(y_{n}-y_{s}{)}^{2}}$$

(3) $$h(n)=\max\left(\left|x_{t}-x_{n}\right|,\left|y_{t}-y_{n}\right|\right)$$

In the formula, $\left(x_{s},{\;y}_{s}\right)$ represents the co-ordinates of the start point, $\left(x_{n},\;y_{n}\right)$ represents the co-ordinates of the current node n, and $\left(x_{t},\;y_{t}\right)$ represents the co-ordinates of the target point.

### 2.2. Improved A* Algorithm

#### 2.2.1. Improved Heuristic Function

According to the principle of the A* algorithm, the search performance mainly depends on the heuristic function h(n). The A* algorithm has different planning effects when using different heuristic functions. The assumption that r(n) is the real cost value from the current node to the target point. When h(n)=0, the A* algorithm degenerates to the Dijkstra algorithm. When searching the path, the algorithm traverses all the nodes, which ensures that the optimal path is found, but wastes most of the time searching for invalid nodes, making the search inefficient. When h(n)<r(n), the search range will be expanded to produce too many extended nodes during the path search, so the path search time is long and less efficient. When h(n)>r(n), the search scope of the algorithm is small, the extended node moves rapidly towards the target point. In this case, although the algorithm’s path search is very fast, it is difficult to plan the optimal path. When $h\left(n\right)=r(n)$, this is the ideal state, and the algorithm will find an optimal path with high search efficiency. To sum up, if we want the A* algorithm to plan an optimal path with high efficiency, we need to pick a suitable heuristic function.

In order to make the heuristic function h(n) closer to r(n), the distance weight factor and logarithmic attenuation factor are introduced to dynamically weight the heuristic function.

The improved heuristic function $h^{\prime }(n)$ is:

(4) $$h^{\prime }(n)=(1+\frac{d}{D})\mathrm{lg}(h(n)+1)h(n)$$

In the formula, d is the Euclidean distance from the current node n to the target point, and D is the Euclidean distance from the starting point to the target point.

Near the starting point, the distance weight factor $\left(1+\frac{d}{D}\right)$ approaches to 2. With the progress of the pathfinding process, the distance weight factor decreases gradually. When near the end point, the distance weight factor $\left(1+\frac{d}{D}\right)$ approaches to 1. According to the characteristics of the logarithmic function, the value of h(n) near the starting point is larger, and the logarithmic attenuation factor $\mathrm{lg}(h\left(n\right)+1)$ is also larger. With the progress of the pathfinding process, h(n) decreases gradually, and the logarithmic attenuation factor $\mathrm{lg}(h\left(n\right)+1)$ decreases gradually. Through the dynamic weight of the distance weight factor and logarithmic attenuation factor to the heuristic function, the heuristic function h(n) accounts for a large proportion in the early stage of path planning, so it can reduce the number of search nodes and make the path move towards the target point faster. When the path is close to the target point, the weight of the heuristic function h(n) is small, so it can expand the search range of nodes and ensure the optimality of the path.

The improved cost function is:

(5) $$f^{\prime }(n)=g(n)+h^{\prime }(n)$$

#### 2.2.2. Path Optimization Strategy

The A* algorithm produces many redundant nodes and turning points during path planning. AGVs will slow down when turning, so the path with more turning points will reduce the efficiency of AGVs. To address this problem, we propose a path optimization strategy in this paper.

**Figure 1:**
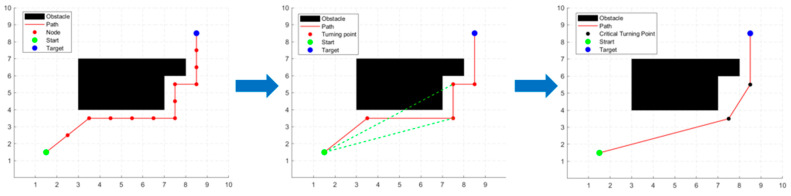
The process of the path optimization strategy.

The process of the path optimization strategy is shown in Figure 1. To remove redundant nodes and turning points, we need to find redundant nodes and turning points in the path first. The redundant nodes are found by judging whether the child node and the parent node of the current node are collinear. The redundant turning points are found by judging whether the lines of the turning points pass through the obstacle. The specific steps are as follows:

Step 1: Remove redundant nodes. Let the set of path nodes planned by the improved A* algorithm be $\left\{Q_{i}|i=1,2,\ldots ,n\right\}$. $Q_{1}$ and $Q_{n}$ represent the starting point and target point respectively. Starting from the second node $Q_{2}$ of the path, it is determined whether $Q_{2}$ is collinear with its parent node $Q_{1}$ and its child node $Q_{3}$. If they are collinear, then node $Q_{2}$ is redundant, delete node $Q_{2}$ and change the parent of node $Q_{3}$ to $Q_{1}$, updating the OPEN list. If they are not collinear, node $Q_{2}$ is preserved. Then, check the nodes $Q_{3}$, $Q_{4}$, …, $Q_{n-1}$ in the same way. Finally, we obtain a new set of path nodes.

Step 2: Remove redundant turning points. After the path is optimized by step 1, the redundant nodes are removed, and then the new path is optimized in the next step to remove redundant turning points. Let the new path nodes set be $\left\{N_{i}|i=1,2,\ldots ,n\right\}$. First, connect node $N_{1}$ to $N_{3}$, $N_{4}$, …, $N_{n}$ in turn to determine whether the straight line crosses the obstacle or not. If the straight line $N_{1}N_{k}(k=3,4,5,\ldots ,n)$ is the line that crosses the obstacle for the first time, then $N_{k-1}$ is the critical turning point, and the nodes between node $N_{1}$ and node $N_{k-1}$ are the redundant turning points, then the redundant turning points are removed and the set of path nodes is updated. Then, connect the node $N_{k}$ to the following nodes in turn, and repeat the above determination step until it is connected to the target point $N_{n}$.

By removing redundant nodes and turning points, the turning angle and path length of the global path can be reduced, which makes the fusion algorithm more stable in tracking the global path and improves the efficiency of path planning of the fusion algorithm.

## 3. Local Path Planning Based on Dynamic Window Method

The dynamic window method is commonly utilized for local path planning [13]. This algorithm can help AGVs to avoid moving obstacles and unknown static obstacles in the working environment. The implementation process of the dynamic window method is: firstly, multiple groups of velocities are sampled in the velocity space of a moving AGV. Secondly, multiple groups of motion trajectories of an AGV in the next interval is simulated according to multiple groups of sampled velocities. Thirdly, the optimal trajectory of the AGV is selected by evaluating the multiple groups of trajectories obtained from the evaluation function. Finally, the velocity corresponding to the optimal trajectory is taken as the velocity instruction of the AGV [16].

### 3.1. The Kinematics Model of AGV

The dynamic window method simulates the trajectories of the next moment based on the sampled velocities, so it is essential to model the kinematics of the AGV. The movement trajectory of the AGV is composed of arc trajectories generated in each sampling interval Δt, because Δt is very short, so each arc trajectory can be regarded as a straight line trajectory. It is assumed that the AGV moves at a constant speed in Δt, the kinematic model of the AGV can be represented as:

(6) $$\begin{array}{c} x_{t+1}=x_{t}+v_{x}\Delta t\cos\theta_{t}-v_{y}\Delta t\sin\theta_{t}\\ y_{t+1}=y_{t}+v_{y}\Delta t\sin\theta_{t}+v_{x}\Delta t\cos\theta_{t}\\ \theta_{t+1}=\theta_{t}+\Delta t\omega \end{array}$$

In the formula, ($x_{t}$, $y_{t}$) and ($x_{t+1}$, $y_{t+1}$) are the position co-ordinates of AGV at time t and time t+1, respectively. $\theta_{t}$ and $\theta_{t+1}$ are the heading angles of AGV a at time t and time t+1, respectively. $v_{x}$ and $v_{y}$ are projections of the running speed of AGV on the *x*-axis and *y*-axis, respectively.

### 3.2. Velocity Sampling

According to the established AGV kinematic model, the trajectories of the AGVs at the next moment can be obtained from the sampled linear and angular velocities. Since there are many sets of velocities to choose from in the velocities space, the sampling velocities need to be constrained according to the conditions of the AGV and the limitations of the environment.

(1) The linear velocity and angular velocity constraints of AGV:

(7) $$V_{m}=\left\{\left(v,\omega )|v\in [v_{\min},v_{\max}],\omega \in [\omega_{\min},\omega_{\max}\right]\right\}$$

In the formula, $v_{min}$ and $v_{max}$ are the minimum and maximum linear velocity of AGV respectively. $\omega_{min}$ and $\omega_{max}$ are the minimum and maximum angular velocity of AGV respectively.

(2) During the time interval Δt, due to the limitations of motor performance and braking performance, the operating velocity of the AGV is constrained to a specified scope, and the constraint is denoted as:

(8) $$V_{d}=\left\{\left(v,\omega )|v\in [v_{c}-{\dot{v}}_{a}\Delta t,v_{c}+{\dot{v}}_{b}\Delta t],\omega \in [\omega_{c}-{\dot{\omega }}_{a}\Delta t,\omega_{c}+{\dot{\omega }}_{b}\Delta t\right]\right\}$$

In the formula, $v_{c}$ and $\omega_{c}$ are the linear velocity and angular velocity of AGV at the current moment. ${\dot{v}}_{a}$ and ${\dot{\omega }}_{a}$ are the maximum linear deceleration and angular deceleration of AGV. ${\dot{v}}_{b}$ and ${\dot{\omega }}_{b}$ are the maximum linear acceleration and angular acceleration of AGV.

(3) For the safe operation of AGVs, it is necessary to ensure that the AGV reduces its velocity to zero before it collides with an obstacle, and the velocity constraints of AGVs is as follows:

(9) $$V_{a}=\left\{(v,\omega )|v\leq \sqrt{2\cdot dist(v,\omega )\cdot {\dot{v}}_{b}},\omega \leq \sqrt{2\cdot dist(v,\omega )\cdot {\dot{\omega }}_{b}}\right\}$$

In the formula, dist(v,ω) denotes the nearest safe distance to an obstacle on the AGV trajectory projected at velocity (v,ω).

### 3.3. Evaluation Function

From the implementation process of dynamic window method introduced earlier, it is known that a suitable evaluation function needs to be designed to evaluate the trajectories simulated by many sets of sampling velocities in the velocities space, then select an optimal trajectory as the next trajectory of AGV. In this paper, we aim to design the evaluation function to ensure that the AGV reaches the target point at a faster speed while maintaining a safe distance from obstacles during operation. The evaluation function is given below:

(10) G(v,ω)=σ(α⋅head(v,ω)+β⋅dist(v,ω)+γ⋅velocity(v,ω))

In the formula, head(v,ω) is used to guide the direction of AGV movement and prevent it from deviating from the target point. dist(v,ω) makes AGV keep a safe distance from the obstacles and prevent collision. velocity(v,ω) makes AGV move to the target point quickly. α, β, γ are the weighting coefficients. σ is the smoothing factor.

## 4. Fusion of Improved A* Algorithm and Dynamic Window Method

The improved A* algorithm can plan a globally optimal path for the AGV, but it cannot cope with dynamic obstacles and unknown static obstacles that may occur in the working environment. Although the dynamic window method is not applicable to global path planning, it has good local obstacle avoidance performance. Therefore, this paper integrates two algorithms to achieve global path planning and dynamic obstacle avoidance. The specific integration steps are as follows:

(1) Planning global paths by improving the A* algorithm.
(2) Optimizing global path using the path optimization strategy.
(3) The critical turning points in the globally optimal path are made as temporary target points for the dynamic window method.
(4) Local path planning between critical turning points using the dynamic window method.
(5) Generate a global optimal path.

**Figure 2:**
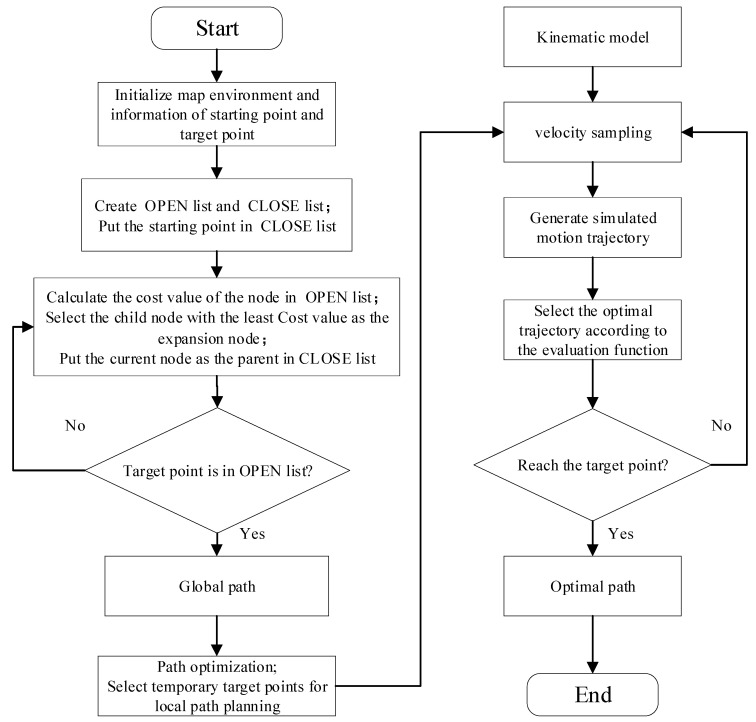
Flow chart of the fusion algorithm.

The fusion algorithm flow is shown in Figure 2.

## 5. Simulation Experiment and Analysis

**Figure 3:**
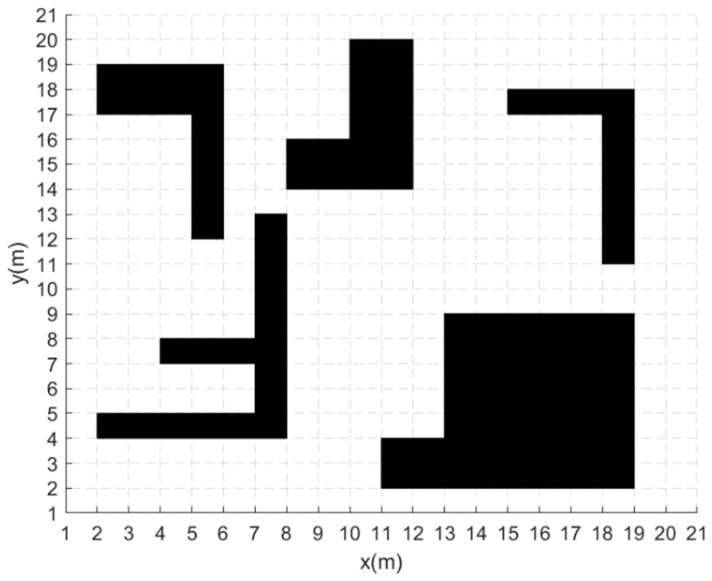
Grid map.

In order to validate the feasibility of the improved A* algorithm and the fusion algorithm, simulation experiments are conducted using MATLAB2020a. The processor of the test host is Intel(R) Core(TM) i5-10400F, the main frequency is 2.90 GHz, and the memory is 16 GB. The working environment of the AGV is simulated using a 20 m×20 m grid map. The grid map is shown in Figure 3, where each grid is 1 m long, the black areas represent the obstacles area and the white areas represent the space where the AGV can move freely. The start point of the AGV is (1.5 m,1.5 m) and the target point is (19.5 m,19.5 m).

The parameters of the dynamic window method are as follows: the maximum linear velocity of the AGV is 1.5 m/s, the maximum linear acceleration is 1 $m/s^{2}$, the maximum angular velocity is 40 rad/s, the maximum angular acceleration is 50 $rad/s^{2}$, the linear velocity resolution is 0.05 m/s, and the angular velocity resolution is 1 rad/s. The parameters of the evaluation function are set as follows: σ=1, α=0.05, β=0.2, γ=0.3, and the prediction time is 2 s.

### 5.1. Simulation Analysis of Heuristic Function

**Figure 4:**
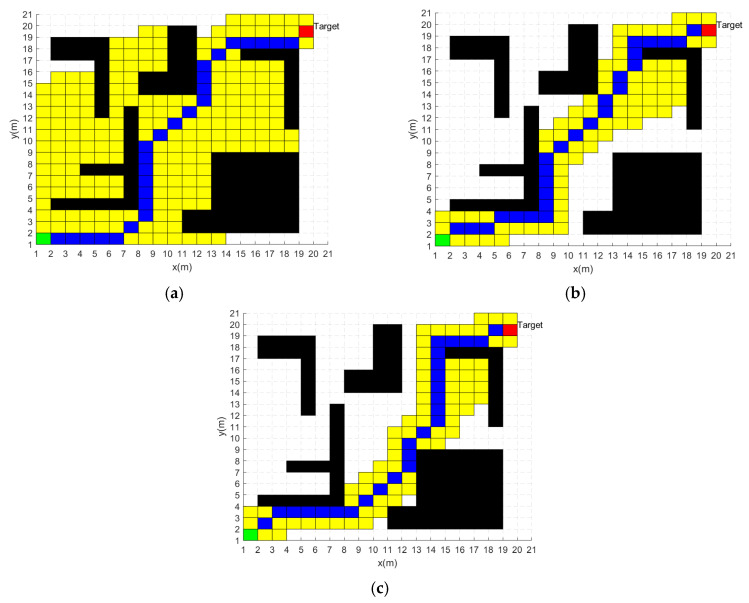
Path planning results of three A* algorithms: (**a**) Traditional A* algorithm. (**b**) Modified A* algorithm. (**c**) Improved A* algorithm.

The traditional A* algorithm has a large search scope and a long search time, resulting in inefficient path planning. After the improvement of the heuristic function, the advantages of the improved A* algorithm in this paper are reflected through comparative analysis of simulation experiments. The simulation results are shown in Figure 4, where the black grids represent the obstacles, the green grid and the red grid represent the starting point and the target point, respectively, the blue grids represent the planning path, and the yellow grids are the search areas of the algorithm. To prevent experimental errors attributed to fluctuations in host performance, the experimental data results are the average values obtained from 20 consecutive simulation experiments.

Figure 4a shows the path-planning results of the traditional A* algorithm. According to the results of simulation experiments, the path planning of the traditional A* algorithm takes 0.076 s, the planned path has a length of 30.73 m, and the search scope occupies 228 $m^{2}$.

Figure 4b shows the path-planning results of the the improved A* algorithm in reference [7]. For ease of representation, the algorithm will be given the name modified A* algorithm. According to the results of simulation experiments, the path planning of the modified A* algorithm takes 0.076 s, the planned path has a length of 30.73 m, and the search scope occupies 228 $m^{2}$.

Figure 4c shows the path planning results of the traditional A* algorithm. According to the results of simulation experiments, the path planning of the improved A* algorithm takes 0.057 s, the planned path has a length of 30.73 m, and the search scope occupies 96 $m^{2}$.

Table 1 summarizes the experimental data for the three A* algorithms. From the above experimental data, it can be seen that all three algorithms have the same planning path lengths. However, the path-planning time of the improved A* algorithm is 26.3% less than the traditional A* algorithm and 4% less than the modified A* algorithm. The improved A* algorithm has 57.9% less search scope than the traditional A* algorithm and 6.6% less search scope than the modified A* algorithm. Therefore, the improved A* algorithm provides some improvement in both search time and search scope.

### 5.2. Global Path Planning in Static Environment

**Figure 5:**
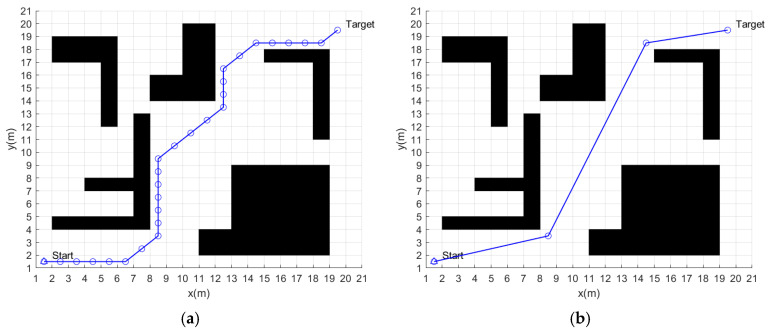

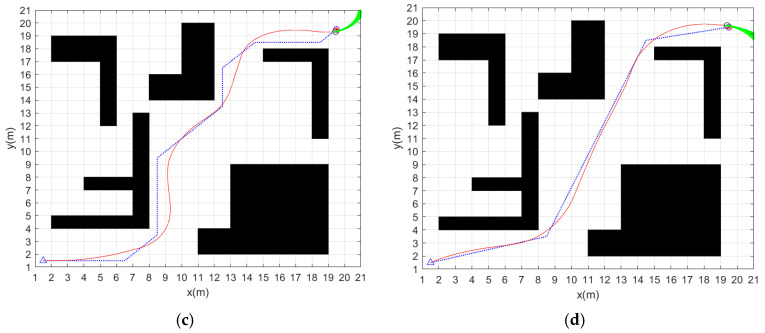
Static global path-planning results of four algorithms: (**a**) Traditional A* algorithm. (**b**) Improved A* algorithm. (**c**) Fusion of traditional A* algorithm and dynamic window method. (**d**) Fusion of improved A* algorithm and dynamic window method.

In a static environment, this paper conducts global path-planning simulation experiments for four algorithms. The simulation images are shown in Figure 5.

The planned path of the traditional A* algorithm is shown in Figure 5a, where the blue circles are the path nodes and the blue line is the path. The time spent on path planning is 0.076 s. The path length is 30.73 m. There are 28 path nodes and seven turning points in the path.

The planned path of the improved A* algorithm is shown in Figure 5b, where the blue circles are the path nodes and the blue line is the path. The time spent on path planning is 0.057 s. The path length is 28.53 m. There are four path nodes and two turning points in the path.

The planned path of the fusion of traditional A* algorithm and dynamic window method is shown in Figure 5c. The blue dashed line and the red solid line are the paths planned by the traditional A* algorithm and the fusion algorithm, respectively. According to the experimental results, the time spent on path planning is 70.56 s and the path length is 30.15 m.

The planned path of the fusion of the improved A* algorithm and dynamic window method is shown in Figure 5d. The blue dashed line and the red solid line are the paths planned by the improved A* algorithm and the fusion algorithm, respectively. According to the experimental results, the time spent on path planning is 57.67 s, and the path length is 28.57 m.

The experimental data of static global path planning for the four algorithms are shown in Table 2.

According to the results of simulation experiments, the path of the improved A* algorithm is 7.2% shorter than the traditional A* algorithm, with 85.7% fewer path nodes and 71.4% fewer turning points.

**Figure 6:**
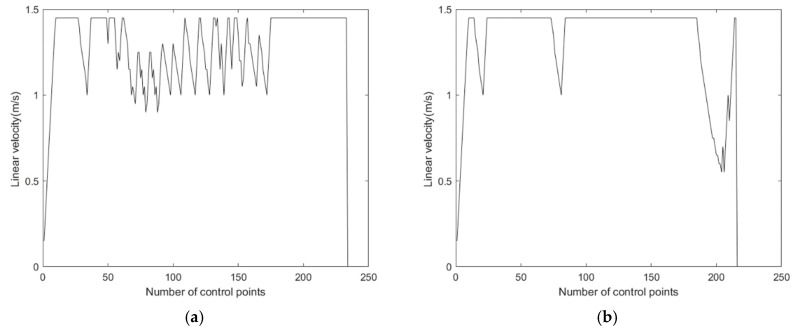
Comparison of linear velocity of two fusion algorithms: (**a**) Fusion of traditional A* algorithm and dynamic window method. (**b**) Fusion of improved A* algorithm and dynamic window method.

**Figure 7:**
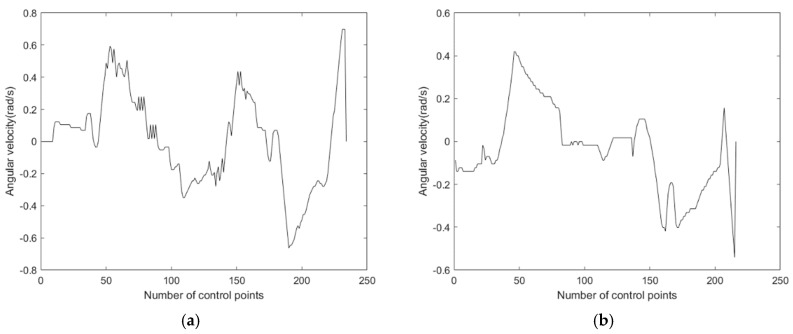
Comparison of angular velocity of two fusion algorithms: (**a**) Fusion of traditional A* algorithm and dynamic window method. (**b**) Fusion of improved A* algorithm and dynamic window method.

The improved A* fusion algorithm reduces the path planning time by 18.3% and the path length by 5.2% compared to the traditional A* fusion algorithm. In addition, Figure 6 and Figure 7 show the linear and angular velocity variations of the AGV during path planning for the two fusion algorithms, respectively. It can be seen from the figures that when the AGV uses the improved A* fusion algorithm to plan the path, the change rate of linear and angular velocity is smaller. It can be seen that the reduction of the number of path nodes and turning points of the improved A* algorithm can help to reduce the path planning time and path length of the fusion algorithm, and can also improve the driving stability of the AGV.

Although the length of the path planned by the fusion algorithm has a slight increase compared to the globally optimal path, the path planned by the fusion algorithm is smoother, which is helpful to improve the motion stability of the AGV.

### 5.3. Global Path Planning in Dynamic Environment

**Figure 8:**
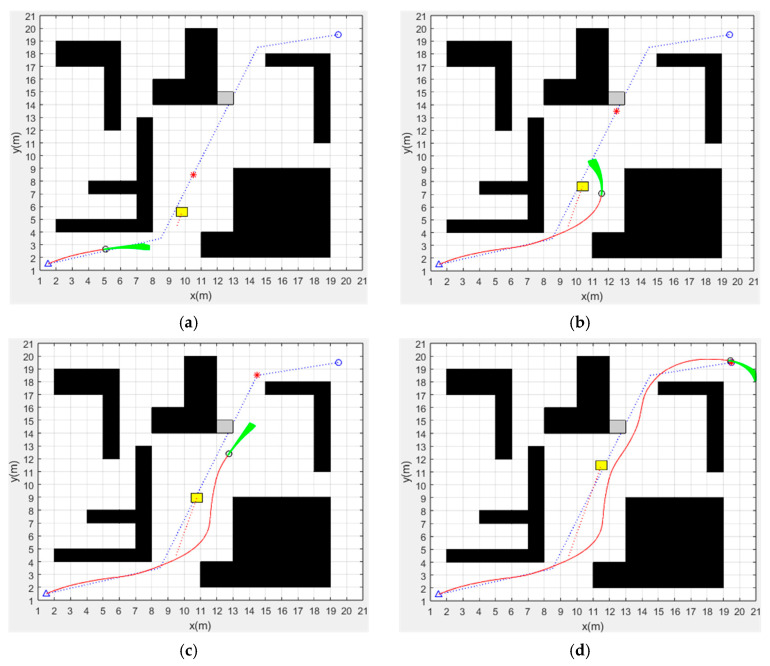
Dynamic path planning of fusion algorithm in map 1: (**a**) Starting path planning. (**b**) Avoiding dynamic obstacle. (**c**) Avoiding unknown static obstacle. (**d**) Reaching the target point.

**Figure 9:**
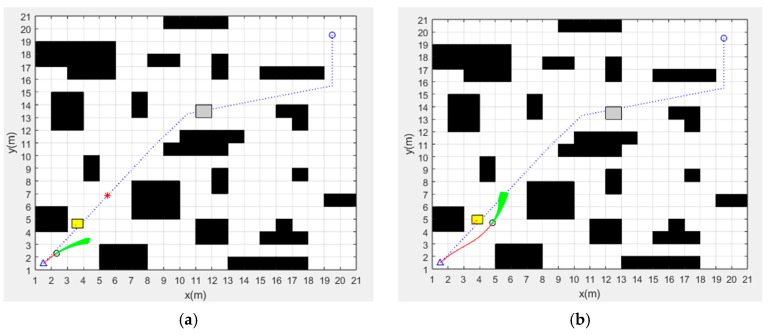

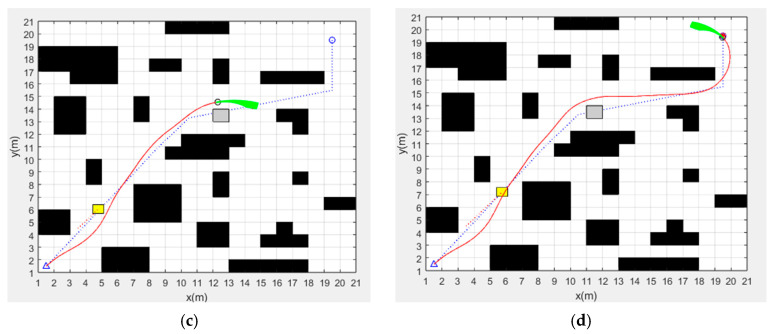
Dynamic path planning of fusion algorithm in map 2: (**a**) Starting path planning. (**b**) Avoiding dynamic obstacle. (**c**) Avoiding unknown static obstacle. (**d**) Reaching the target point.

**Figure 10:**
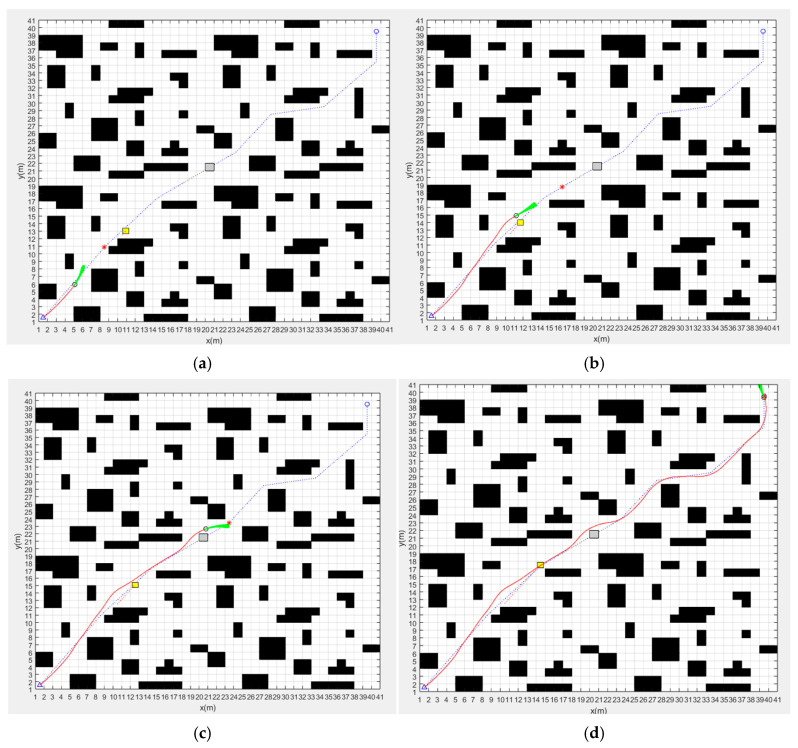
Dynamic path planning of fusion algorithm in map 3: (**a**) Starting path planning. (**b**) Avoiding dynamic obstacle. (**c**) Avoiding unknown static obstacle. (**d**) Reaching the target point.

In order to verify the obstacle-avoidance performance of the fusion algorithm and its sensitivity to environmental changes, we have performed dynamic obstacle-avoidance simulation experiments in different map environments. The path planning effect of the fusion algorithm in map 1, map 2 and map 3 is shown in Figure 8, Figure 9 and Figure 10, respectively, The parameters of three different map environments are shown in Table 3. In addition to the known obstacles in the map, we add dynamic obstacles and unknown static obstacles to the possible path of the AGV to investigate the dynamic obstacle-avoidance ability of the fusion algorithm. In the figures, black squares represent known static obstacles, yellow squares represent dynamic obstacles, gray squares represent unknown static obstacles, the blue dotted line is the global optimal path planned by the improved A* algorithm, and the red solid line is the path planned by the fusion algorithm.

The path-planning effect of the fusion algorithm in map 1 is shown in Figure 8. Figure 8a shows that the fusion algorithm starts path planning along the global optimal path. Figure 8b shows that the AGV detects moving obstacle and starts local path planning to successfully avoid moving obstacles and then continues to follow the global optimal path. Figure 8c shows that the AGV detects an unknown static obstacle and starts local path planning to successfully avoid it and then continues to follow the global optimal path. Figure 8d shows that the AGV successfully reached the target point. It can be seen from the figure that the fusion algorithm can successfully avoid dynamic obstacles and unknown static obstacles in the process of following the global optimal path. The simulation results show that the path-planning time of the fusion algorithm in map 1 is 58.76 s and the path length is 29.30 m.

The path-planning effect of the fusion algorithm in map 2 is shown in Figure 9. The simulation results show that the path-planning time of the fusion algorithm in map 2 is 59.20 s and the path length is 28.46 m. As can be seen in the figure, the fusion algorithm can also achieve dynamic obstacle avoidance and reach the target point in the more complex map.

The path-planning effect of the fusion algorithm in map 3 is shown in Figure 10. The simulation results show that the path-planning time of the fusion algorithm in map 3 is 260.82 s and the path length is 57.14 m. From the simulation results, it can be seen that although the path-planning efficiency of the fusion algorithm is reduced in the larger map, it can still satisfy the requirements of a global optimal path and dynamic obstacle avoidance.

From the path-planning results of the fusion algorithm in three different map environments, we can see that the fusion algorithm proposed in this paper can be applied to the global optimal path planning and real-time dynamic obstacle avoidance in different map environments.

## 6. Conclusions

In order to improve the operational efficiency of the AGV in logistics, warehousing and industrial production environments and ensure the safety of AGVs, this paper proposes a global dynamic path-planning fusion algorithm, which combines the improved A* algorithm and dynamic window method. The fusion algorithm has the advantages of high planning efficiency, smooth path and dynamic obstacle avoidance.

(1) In order to make the heuristic function closer to the real cost value r(n), the distance weight factor and logarithmic attenuation factor are used to weight the heuristic function dynamically. This method can provide effective help to reduce the search scope and shorten the path-planning time.
(2) The path-optimization strategy is adopted to remove the redundant nodes and redundant turning points of the planned path, and improve the smoothness of the path. This strategy is beneficial to the stability of AGV driving.
(3) The improved A* algorithm is fused with the dynamic window method to solve the problems of unsmooth paths and inability to realize dynamic obstacle avoidance.
(4) The method proposed in this paper is verified by simulation experiments. The path-planning time of the improved A* algorithm is 26.3% shorter than the traditional A* algorithm, the search scope is 57.9% less, the path length is 7.2% shorter, the number of path nodes is 85.7% less, and the number of turning points is 71.4% less. The fusion of improved A* algorithm and dynamic window method can realize global optimal path planning and local dynamic obstacle avoidance in different map environments.

The deficiency and prospect of this study:

(1) The fusion algorithm in this paper has only carried out simulation experiments and has not been applied to the actual AGV. The next step is to apply the fusion algorithm to AGVs and carry out path planning in the actual environment.
(2) The fusion algorithm in this paper is not combined with environment awareness technology, so we can only carry out path planning in the established map environment. The next step is to integrate environment awareness technology to realize path planning in an unfamiliar environment.

## Figures and Tables

**Table 1:** Experimental data of three A* algorithms.

Algorithm	Path Length/m	Planning Time/s	$\mathrm{Search}\;\mathrm{Scope}{/m}^{2}$
Traditional A* Algorithm	30.73	0.076	228
Modified A* Algorithm.	30.73	0.061	100
Improved A* Algorithm	30.73	0.057	96

**Table 2:** Experimental data of four algorithms.

Algorithm	Planning Time/s	Number of Path Nodes	Number of Turning Points	Path Length/m
Traditional A* algorithm	0.076	28	7	30.73
Improved A* algorithm	0.057	4	2	28.53
Fusion of traditional A* algorithm and dynamic window method	70.56	/	/	30.15
Fusion of Improved A* algorithm and dynamic window method	57.67	/	/	28.57

**Table 3:** The parameters of three different map environments.

Name	Size/m	Start-Target Point Coordinates	Number of Known Obstacles
Map 1	20×20	(1.5, 1.5) (19.5, 19.5)	5
Map 2	20×20	(1.5, 1.5) (19.5, 19.5)	19
Map 3	40×40	(1.5, 1.5) (39.5, 39.5)	74

## Data Availability

Data are contained within the article.
